# Scalp acupuncture and electromagnetic convergence stimulation for patients with cerebral infarction: study protocol for a randomized controlled trial

**DOI:** 10.1186/s13063-016-1611-y

**Published:** 2016-10-11

**Authors:** Jae-Young Han, Jae-Hong Kim, Ju-Hyung Park, Min-Yeong Song, Min-Keun Song, Dong-Joo Kim, Young-Nim You, Gwang-Cheon Park, Jin-Bong Choi, Myung-Rae Cho, Jeong-Cheol Shin, Ji-Hyun Cho

**Affiliations:** 1 Department of Physical and Rehabilitation Medicine, Chonnam National University Medical School and Hospital, 42, Jebong-ro, Dong-gu, Gwangju City, 61469 Republic of Korea; 2 Department of Acupuncture and Moxibustion Medicine, College of Traditional Korean Medicine, Dong-Shin University, 185, Geonjae-ro, Naju City, 58245 Republic of Korea; 3 Clinical Research Center, Gwangju Traditional Korean Medicine Hospital of Dong-Shin University, 141, Wolsan-ro, Nam-gu, Gwangju City, 61619 Republic of Korea; 4 Department of Korean Medicine Rehabilitation, College of Traditional Korean Medicine, Dong-Shin University, 185, Geonjae-ro, Naju City, 58245 Republic of Korea; 5 Department of Social Welfare, College of Health and Welfare, Dong-Shin University, 185, Geonjae-ro, Naju City, 58245 Republic of Korea

**Keywords:** Cerebral infarction, Scalp acupuncture, Repetitive transcranial magnetic stimulation, Collaborative study, Randomized controlled trial, Study protocol

## Abstract

**Background:**

Scalp acupuncture (SA) and repetitive transcranial magnetic stimulation (rTMS) are effective for treating cerebral infarction. This study aims to examine the efficacy and safety of SA and electromagnetic convergence stimulation (SAEM-CS), which was developed through collaboration between conventional medical physicians and doctors who practice traditional Korean medicine. SAEM-CS was designed to improve function in patients with cerebral infarction, compared to the improvement after conventional stroke rehabilitation, SA, and rTMS therapeutic approaches.

**Methods/design:**

This study is a prospective, outcome assessor-blinded, randomized controlled clinical trial with a 1:1:1:1 allocation ratio. Participants with motion or sensory disabilities caused by a first-time cerebral infarction (*n* = 60) that had occurred within 1 month of the study onset will be randomly assigned to control, SA, rTMS, or SAEM-CS groups. All groups will receive two sessions of conventional rehabilitation treatment per day. The SA group will receive SA on the upper limb area of MS6 and MS7 (at the lesional hemisphere) for 20 min, the rTMS group will receive low-frequency rTMS (LF-rTMS) treatment on the hot spot of the M1 region (motor cortex at the contralesional hemisphere) for 20 min, and the SAEM-CS group will receive LF-rTMS over the contralesional M1 region hot spot while receiving simultaneous SA stimulation on the lesional upper limb area of MS6 and MS7 for 20 min. SA, rTMS, and SAEM-CS treatments will be conducted once/day, 5 days/week (excluding Saturdays and Sundays) for 3 weeks, for a total of 15 sessions. The primary outcome will be evaluated using the Fugl‐Meyer Assessment, while other scales assessing cognitive function, activities of daily living, walking, quality of life, and stroke severity are considered secondary outcome measures. Outcome measurements will be conducted at baseline (before intervention), 3 weeks after the first intervention (end of intervention), and 4 weeks after intervention completion.

**Discussion:**

This study aims to explore the efficacy and safety of SAEM-CS on cerebral infarction. Collaborative research combined traditional Korean and conventional medicines, which can be useful in developing new treatment technologies.

**Trial registration:**

KCT0001768. Registered on 14 January 2016.

**Electronic supplementary material:**

The online version of this article (doi:10.1186/s13063-016-1611-y) contains supplementary material, which is available to authorized users.

## Background

Cerebral infarction (CI) is one of the most commonly reported cerebral vascular diseases, accounting for about 70 % of strokes [1]. The incidence, mortality, and recurrence rates of CI are high, and CI usually leads to serious damage of the central nervous system [2]. Despite a considerable amount of research on effective treatments for stroke, there is still no single intervention that clearly and definitively contributes to stroke recovery. Therefore, stroke treatment strategies should combine multiple disciplines, such as neurology, rehabilitation medicine, and traditional medicine [3]. Scalp acupuncture (SA) is one of several specialized acupuncture techniques, and it involves a filiform needle being used to penetrate specific stimulation areas on the scalp [4]. SA therapy for ischemic and hemorrhagic stroke has been empirically established and is used worldwide [5–8]. The local application of repetitive transcranial magnetic stimulation (rTMS) influences the neural excitability of selected brain areas [9], and it has been reported that low-frequency stimulation (1 Hz) suppresses local neural activities [10, 11], whereas high-frequency stimulation (≥5 Hz) activates local neural activities [12]. Both high-frequency rTMS (HF-rTMS) applied to the lesional hemisphere and low-frequency rTMS (LF-rTMS) applied to the contralesional hemisphere are beneficial for upper limb hemiparesis in patients with chronic stroke [11, 13–17] and in the early phase of stroke [9, 18–20].

Conventional medical physicians and doctors who practice traditional Korean medicine (TKM) tend to focus on each field’s treatment methods in separate medical systems. Unfortunately, efforts to improve the treatment rates of incurable diseases through collaborative research and via the fusion of treatment techniques are lacking. The purpose of this study is to explore the efficacy and safety of SA and electromagnetic convergence stimulation (SAEM-CS), which was developed through a collaborative study between the Department of Physical and Rehabilitation Medicine at Chonnam National University Hospital and the Departments of Acupuncture and Moxibustion Medicine and Traditional Korean Medicine Rehabilitation at Dong-Shin University. Thereafter, we will compare SAEM-CS to conventional stroke rehabilitation, SA, and rTMS therapeutic approaches.

## Methods/design

### Objective

The objective of this study is to compare the efficacy of SAEM-CS on motor function recovery to physical therapy, rTMS, and SA. Further, we aim to explore the synergistic effect of converging SA and rTMS, which are known to be effective in promoting the recovery of motor function, in patients with CI.

### Study design

This study is a prospective, outcome assessor-blinded, single-center, randomized controlled clinical trial with a 1:1:1:1 allocation ratio. This study is a pilot study to investigate the efficacy and safety of SAEM-CS, which is a newly developed treatment. This study is designed as a single-center study to overcome several procedural and organizational difficulties in terms of developing a research team, since SAEM-CS involves the simultaneous conduction of SA and rTMS. Participants (*n* = 60) who fit the inclusion and exclusion criteria will be randomly allocated into a control group (*n* = 15), SA group (*n* = 15), rTMS group (*n* = 15), and SAEM-CS group (*n* = 15). All groups will receive conventional stroke rehabilitation therapy twice/day, five times/week (excluding Saturdays and Sundays), for a total of 15 times over the course of a 3-week hospitalization period at Chonnam National University Hospital. In addition, the SA group will receive SA therapy, the rTMS group will receive rTMS therapy, and the SAEM-CS group will receive SAEM-CS therapy once/day. Outcome measures will be determined at baseline (before intervention), 3 weeks after the first intervention (end of the intervention), and 4 weeks after completion of the intervention.

This study was approved by the Ministry of Food and Drug Safety (MFDS), Medical Device Clinical Trial Plan Approval number 516. (See Additional file 1.)

**Fig. 1:**
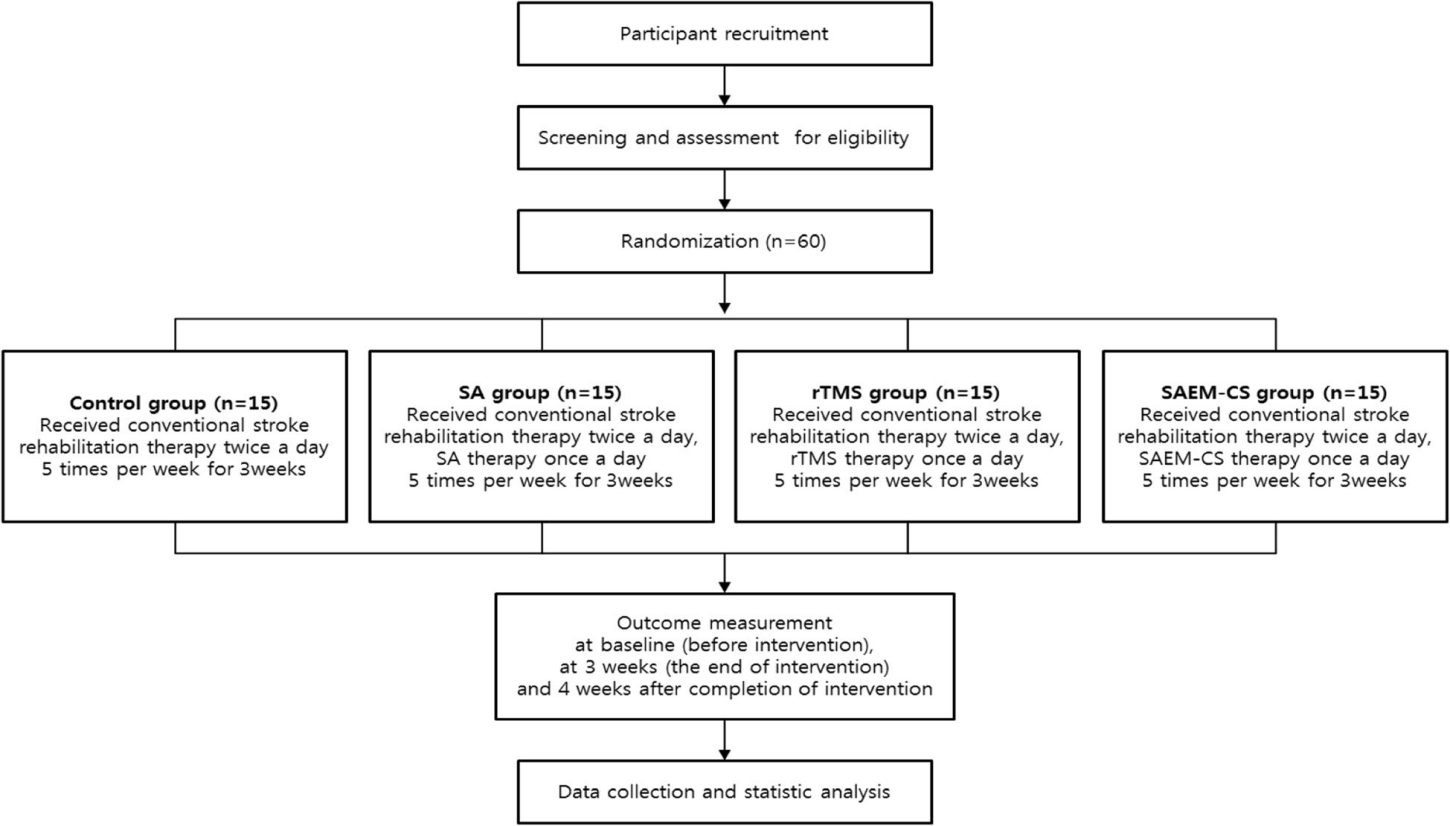
Study design flow chart

The study design is summarized in Table 1 and Fig. 1.

**Table 1:**
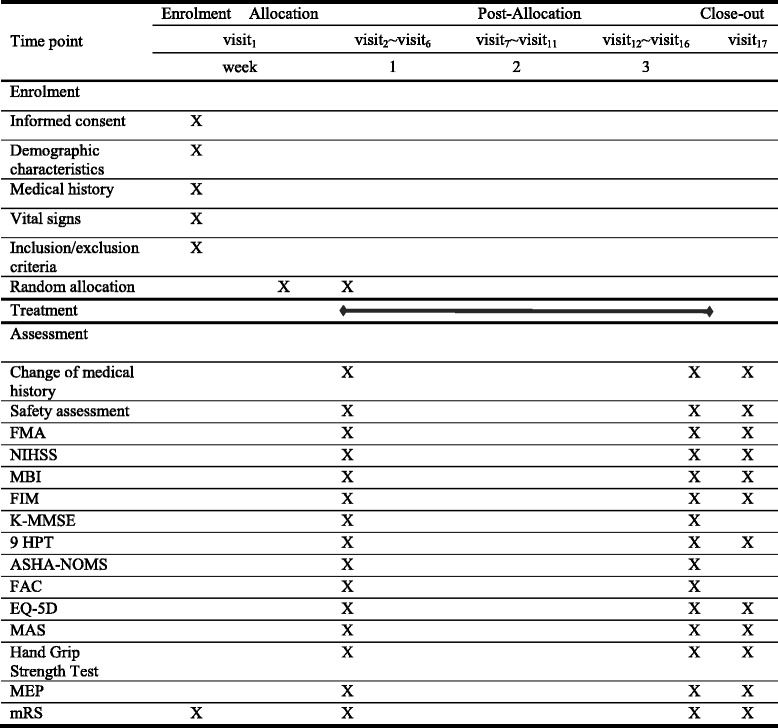
Treatment schedule and outcome measures

*FMA* Fugl-Meyer Assessment, *NIHSS* National Institutes of Health Stroke Scale, *MBI* Modified Barthel Index, *FIM* Functional Independence Measurement, *K-MMSE* Korean Mini-Mental State Examination, *9HPT* 9-Hole Peg Test, *ASHA-NOMS* American Speech-Language-Hearing Association National Outcomes Measurement System, *FAC* Functional Ambulation Categories, *EQ-5D* European Quality of Life-5 Dimensions, *MAS* Modified Ashworth Scale, *MEP* motor evoked potential, *mRS* modified Rankin Scale

### Participant recruitment

For achieving adequate participant enrollment to reach target sample size, all stroke patients admitted to the Department of Neurology of Chonnam National University Hospital will be screened by Physical and Rehabilitation Medicine doctors. From among the first-ever patients with CI who are hospitalized and have finished treatment of early acute stage CI at the Department of Neurology, those who are assessed as stable by a neurologist and meet the inclusion and exclusion criteria will be recruited for participation in this study.

Patients who are given an explanation about this study by the Clinical Research Coordinator (CRC) and who voluntarily sign a consent form will be transferred to the Department of Physical and Rehabilitation Medicine to participate in this study (see Additional file 2). The CRC will continuously monitor the medical conditions of enrolled participants to improve adherence to intervention protocols.

### Inclusion criteria

Patients who meet all of the following conditions will be considered for enrollment: patients who are aged >19 years; who have incipient CI confirmed by a computed tomography or magnetic resonance imaging examination; who are diagnosed by a neurologist or neurosurgeon; who experience a CI that resulted in motor and sensory disorders within 1 month of the study onset; who can undergo rehabilitation therapy after hospitalization in the Department of Physical and Rehabilitation Medicine, Chonnam National University Hospital; who have a modified Rankin Scale (mRS) score of 2–4; and who voluntarily sign an informed consent form.

### Exclusion criteria

Subjects whose general condition is not good or fit for SA and rTMS therapies will be excluded. Additional exclusion criteria are as follows: history of brain lesion (e.g., stroke, serious mental illness, loss of consciousness accompanied by head trauma, brain surgery, or seizure disorder); presence of other serious illnesses (e.g., cancer, Alzheimer’s disease, epilepsy, head trauma, or cerebral palsy); transient ischemic attacks; contraindications to electro-magnetic stimulation (e.g., metal implants in the brain, implanted electronic devices in the body, such as non-detachable ferromagnetic metals, metal-sensitive implants less than 30 cm away from the brain like cochlear implants, pacemakers, aneurysm clips or coils, stents, bullet fragments, deep brain stimulation, vagus nerve stimulators, jewelry, or hairpins); continuous convulsion symptoms; previous craniectomy or shunt surgery; elevated intracranial pressure symptoms such as headache, vomiting, nausea, etc.; seizure disorder or epilepsy after CI; history of stroke accompanied by a clear clinical sign; contraindications to SA (e.g., scalp scarring, inflammation from scalp injury, or infection in the treatment region, inability to stop blood flow due to clotting disturbances, such as hemophilia, etc., serious unusual response after acupuncture treatment); women who are pregnant or breastfeeding; patients who disagree with the informed consent; and individuals scheduled for surgery within 2 weeks.

### Ethical considerations

The Institutional Review Board of Chonnam National University Hospital approved this study (CNUH-2015-114; see Additional file 3). The purpose and potential risks of this clinical trial will be fully explained to the patients and their families. All patients will be asked to provide written informed consent before participating in this study.

### Randomization

After signed informed consent and baseline measurements are obtained, random allocation software (developed by M. Saghaei, M.D., at the Department of Anesthesia, Isfahan University of Medical Sciences, Isfahan, Iran) is used to assign a serial number to the 60 research volunteers and to randomly allocate 15 of them into each group. The serial number codes will be inserted into sealed, opaque envelopes, kept in a double-locked cabinet, and opened in the presence of the patient and a guardian.

### Implementation

A CRC will generate the allocation sequence, enroll participants, and assign participants to interventions.

### Blinding

We have no choice but to adopt a single blinding (outcome assessor blinding) approach, as sham treatment — where participants and practitioners are not aware of the treatment condition — is impossible due to the characteristics of scalp acupuncture which include scalp penetration. Efforts to maintain objectivity are made by separating the CRC who manages the treatment schedules, the practitioner who conducts the treatments, and the assessor. During the course of this clinical trial, the assessor will not come into contact with any of the participants except at the time of assessment. Further, there is no circumstance where unblinding will be permitted. To prevent risks of bias, such as selection, performance, and attrition caused by non-blinding of participants and practitioners, only individuals without conflicts of interest or preconceived positions are involved in this study. All practitioners will receive training in clinical trials prior to participation in this study.

### Intervention

All participants will receive occupational therapy, which focuses on practicing fine and gross motor movements, activities of daily living, task-oriented therapeutic exercises, and muscular electrical stimulation therapy, as needed. Training for swallowing and to improve language will be also performed for dysarthria. These sessions will be conducted for 30 min, twice daily (excluding Saturdays and Sundays) for 3 weeks, for a total of 15 times. SA, rTMS, and SAEM-CS therapies will be conducted once daily for 20 min (excluding Saturdays and Sundays) for 3 weeks, for a total of 15 times (see Table 2).

**Table 2:** Three-week treatment protocol (example for a patient admitted on a Monday)

		Monday–Friday	Saturday, Sunday	Monday–Friday	Saturday, Sunday	Monday–Friday		Four weeks after intervention completion
Morning	Admission Assessment at baseline	Physical therapy (30 min)	No treatment	Physical therapy (30 min)	No treatment	Physical therapy (30 min)	Discharge Assessment at the end of intervention	Follow-up assessment
		Occupational therapy (30 min)		Occupational therapy (30 min)		Occupational therapy (30 min)		
		Functional electrical stimulation (30 min)		Functional electrical stimulation (30 min)		Functional electrical stimulation (30 min)		
Afternoon		Physical therapy (30 min)		Physical therapy (30 min)		Physical therapy (30 min)		
		Occupational therapy (30 min)		Occupational therapy (30 min)		Occupational therapy (30 min)		
		Functional electrical stimulation (30 min)		Functional electrical stimulation (30 min)		Functional electrical stimulation (30 min)		
		SA group: SA therapy rTMS group: rTMS therapy SAEM-CS group: SAEM-CS therapy		SA group: SA therapy rTMS group: rTMS therapy SAEM-CS group: SAEM-CS therapy		SA group: SA therapy rTMS group: rTMS therapy SAEM-CS group: SAEM-CS therapy		

The SA therapy method will be conducted as follows: one or two needles are horizontally inserted about 3 cm deep at the lesion site and upper limb regions of MS6 (line connecting GV21 and GB6) and MS7 (line connecting GV20 and GB7) [8] under the Standard International Nomenclature in the direction from GV21 to GB6 and GV20 to GB7. Manual stimulation and electroacupuncture are not applied, and the needles (KOS-92 non-magnetic steel acupuncture needles; size 0.25 mm × 30 mm; Dong Bang Acupuncture, Inc., Boryeong, Republic of Korea; Product no: A84010.02) (see Table 3) are left in position for 20 min (see Table 4).

**Table 3:** KOS-92 alloy distribution

Type	C	Si	Mn	P	S	Ni	Cr	N	Mo	Other	Note
STS304	0.08	1.0	2.0	0.045	0.03	8–10.5	18–20				
STS304N1	0.08	1.0	2.5	0.045	0.03	8–10.5	18–20	0.1–0.25			
STS316	0.08	1.0	2.0	0.045	0.03	10–14	16–18		2–3		
KOS-92	0.08	1.0	10	0.03	0.03	5–6	17–18	0.25–0.35			Non-magnetic

Unit: %

**Table 4:** Revised Standards for Reporting Interventions in Clinical Trials of Acupuncture (STRICTA)

	Item criteria	Description
1. Acupuncture rationale	1a) Style of acupuncture	Korean medicine therapy
	1b) Reasoning for treatment provided, based on historical context, literature sources, and/or consensus methods, with references where appropriate	1) Discussion among four doctors who practice Korean medicine (consensus) 2) Textbook of acupuncture and moxibustion medicine 3) Relevant articles [7, 8] Selection of treatment regions based on textbooks, related papers, and expert discussions
	1c) Extent to which treatment varied	Standardized treatment
2. Details of needling	2a) Number of needle insertions per subject per session (mean and range where relevant)	2–4
	2b) Names (or location if no standard name) of points used (uni-/bilateral)	SIAN’s MS6; MS7 of the lesional hemisphere
	2c) Depth of insertion, based on a specified unit of measurement or on a particular tissue level	Needles were horizontally inserted into the subcutaneous tissue of the scalp, about 3 cm deep.
	2d) Responses sought	No de qi or muscle twitching — only sensation due to needle insertion
	2e) Needle stimulation	None
	2f) Needle retention time	20 min per session
	2 g) Needle type	KOS-92 non-magnetic steel disposable needles (0.25-mm diameter and 30-mm length), manufactured by Dong Bang Acupuncture, Inc.
3. Treatment regimen	3a) Number of treatment sessions	15
	3b) Frequency and duration of treatment sessions	Five times/week for 3 weeks, 20 min per session
4. Other treatment components	4a) Details of other interventions administered to the acupuncture group	Conventional stroke rehabilitation therapy
	4b) Setting and context of treatment, including instructions to practitioners, as well as information and explanations given to patients	Practitioner-patient conversation about the context of the treatment, life habits, and daily life management
5. Practitioner background	5) Description of participating acupuncturists	Korean medicine doctor with the following qualifications: 6 years of formal university training in Korean medicine, a license, and at least 2 years of clinical experience
6. Control or comparator interventions	6a) Rationale for the control or comparator in the context of the research question, with sources that justify the choice	Wang Y, Shen J, Wang XM, Fu DL, Chen CY, Lu LY, et al. Scalp acupuncture for acute ischemic stroke: a meta-analysis of randomized controlled trials. *Evid Based Complement Altern Med* 2012;2012:480950; Lee SJ, Shin BC, Lee MS, Han CH, Kim JI. Scalp acupuncture for stroke recovery: a systematic review and meta-analysis of randomized controlled trials. *European J Integr Med*. 2013;5:87–99
	6b) Precise description of the control or comparator; details for items 1–3 above with the use of sham acupuncture or any other type of acupuncture-like control	Conventional stroke rehabilitation therapy for control, rTMS, and SAEM-CS groups. LF-rTMS applied to the hot spot of the M1 region (the motor cortex at the contralesional hemisphere) for the rTMS group and LF-rTMS applied to the same M1 and simultaneous SA stimulation over the upper MS6 and MS7 regions of the lesional hemisphere for the SAEM-CS group

The rTMS method will be conducted as follows: a 70-mm figure 8 coil and a Magstim Rapid stimulator (Magstim Co., Dyfed, UK) is used to deliver 1 Hz of rTMS to the skull of the contralesional hemisphere at the site that elicits the largest motor evoked potentials (MEPs) in the first dorsal interosseous (FDI) muscle of the unaffected upper limb. One LF-rTMS session consists of 1200 pulses and lasts for 20 min. The stimulation intensity is set to 80 % of the motor threshold of the FDI muscle, which is defined as the lowest intensity of stimulation that provokes MEPs [15, 16]. All patients sit in a reclining wheelchair and are asked to relax as much as possible, with their heads strapped to a headrest [9].

**Fig. 2:**
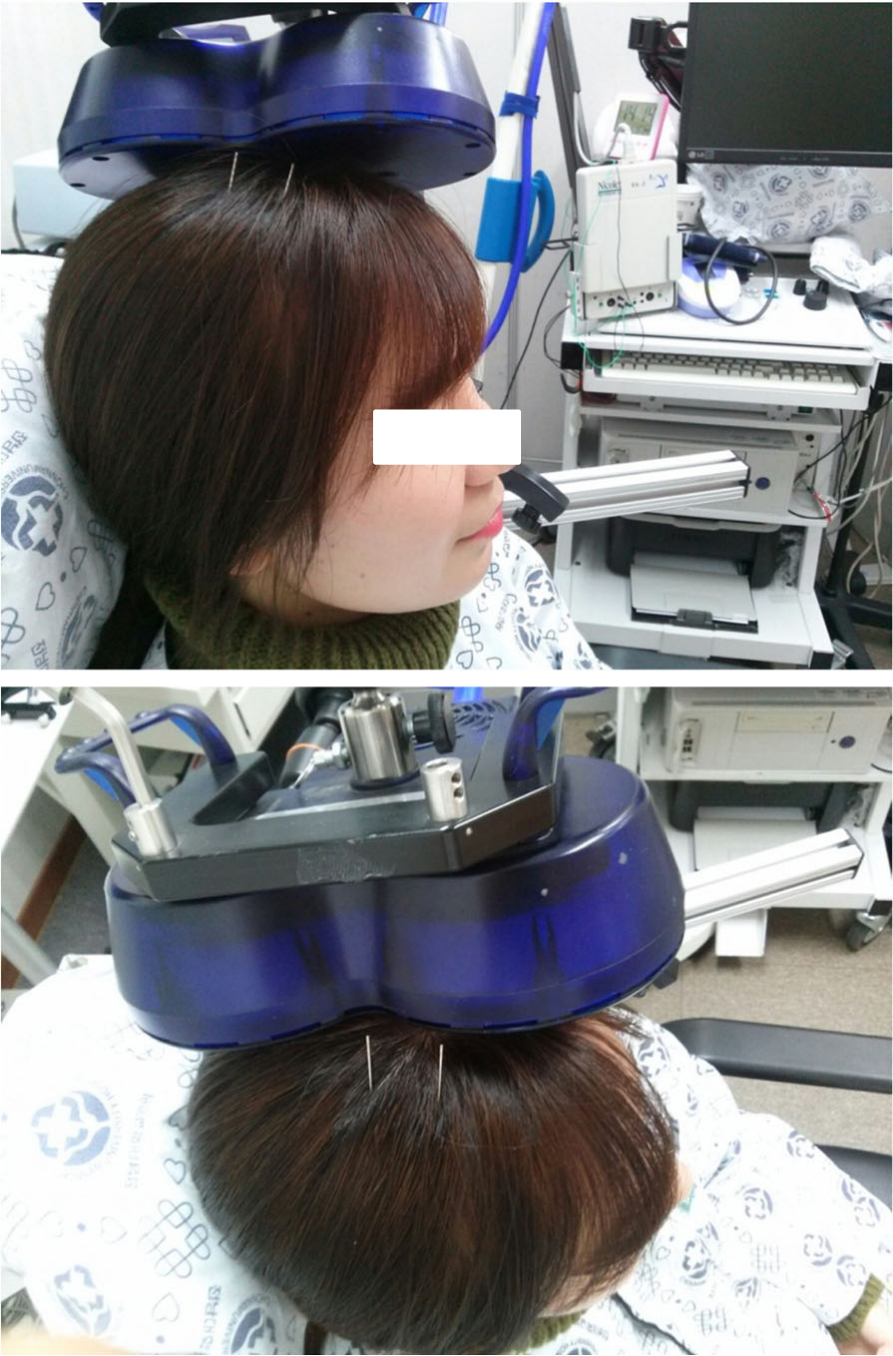
Application of SAEM-SC. LF-rTMS over the M1 region hot spot (motor cortex at the left hemisphere) and SA stimulation of MS6 and MS7 at the upper limb regions of the right hemisphere

The SAEM-CS treatment method will be conducted as follows: the above-described SA and LF-rTMS therapies are performed simultaneously. After SA treatment of MS6 and MS7 on the lesion side, LF-rTMS stimulation is conducted on the contralateral hemisphere of the opposite side of the lesion for 20 min (Fig. 2).

The SA therapy will be conducted by a doctor who is experienced in Korean medicine, is affiliated with Dong-Shin Traditional Korean Medicine University Hospital, and has more than 2 years of clinical experience. rTMS will be conducted by a doctor affiliated with the Department of Physical and Rehabilitation Medicine at Chonnam National University Hospital.

During the clinical trial period, all participants are allowed routine management, existing medications (medications for hypertension, diabetes, hyperlipidemia, and improvement of brain function), and medications to maintain and improve health status. However, patients are not allowed to engage in treatments to improve CI other than the therapies used in this study.

All medical devices will be inspected by coordinators who will manage scalp acupuncture needles (Min-yeong Song, Resident of Department of Korean Medicine Rehabilitation, College of Traditional Korean Medicine, Dong-Shin University) and rTMS (Eom-ji Kim, occupational therapist, Department of Physical and Rehabilitation Medicine at Chonnam National University Hospital). Coordinators will record the results of the check-ups in the management register.

### Outcome measurements

Primary and secondary outcome assessments will be conducted at baseline (before intervention), 3 weeks after the first intervention, and 4 weeks after completion of intervention.

### Primary outcome

Since the objective of this study is to investigate the efficacy of SAEM-CS on motor function recovery in patients with cerebral infarction, the primary outcome will be assessed via changes on the Fugl‐Meyer Assessment (FMA) scale for motor function. The FMA is widely used as a qualitative measure of motor function and is used in the current study to assess upper and lower limb function. The FMA is categorized into 50 items based on a six-stage recovery process of Brunnstrom’s hemiplegia classification and progress record. This assessment is an ordinal scale, in which 0 is given for unable to perform, 1 for partial performance, and 2 for complete performance. The FMA has a possible total score of 100, 66 points of which apply to the upper limbs and 34 points to the lower limbs [16].

### Secondary outcomes

Secondary outcome measures will be assessed changes in the National Institutes of Health Stroke Scale (NIHSS) score, Modified Barthel Index (MBI), Functional Independence Measurement (FIM) score, Korean Mini-Mental State Examination (K-MMSE) score, American Speech-Language-Hearing Association National Outcomes Measurement System (ASHA-NOMS) Swallowing Scale score, Functional Ambulation Categories (FAC), European Quality of Life-5 Dimensions (EQ-5D), Modified Ashworth Scale (MAS) score, Hand Grip Strength Test, MEPs, mRS score, and 9-Hole Peg Test (9HPT).

The NIHSS is a scale that was developed by the United States National Institutes of Health, and provides useful information on the severity, prognosis, and early treatment of stroke [9].

The MBI is an assessment tool used to objectively evaluate performance of everyday movements on 10 items: personal hygiene, bathing, eating, toilet use, ability to climb stairs, ability to dress, bowel/bladder function, walking function, capability of getting into and out of a chair/bed, and mobility [21].

The FIM is an assessment of everyday movement performance that evaluates 13 detailed items of motor FIM and 5 detailed items of cognitive FIM. The FIM has a total possible score of 18–126, and each item is divided into seven stages, allowing for a comparably detailed assessment. Higher FIM scores indicate higher functional independence [22].

The MMSE is a brief global instrument used to assess cognitive abilities and has been translated into Korean (K-MMSE) [23]. The K-MMSE is a quick screening assessment tool that was developed to evaluate cognitive function and dementia in elderly patients with suspected dementia or patients with brain damage. The instrument consists of measures used to assess orientation, recall, attention and calculation, language, judgment, and understanding. A score ≥24 points indicates definitive normal, 20–23 indicates suspected dementia, and ≤19 indicates definitive dementia [24].

The ASHA-NOMS is a seven-stage dysphagia scale developed by the American Speech-Language-Hearing Association to evaluate severity of dysphagia for items such as parenteral hyperalimentation, whether independent eating is possible, use of compensative skills for eating, and diet limitations [25].

The FAC is designed to evaluate walking ability where a patient’s independent walking ability is categorized into six ranks: 0 = unable to walk, 1 = direct assistance required by the therapist, 2 = intermittent and direct assistance required by the therapist, 3 = able to walk independently but requires observation, 4 = able to walk independently but requires assistance on uneven surfaces, and 5 = able to independently walk on even surfaces [26].

The EQ-5D evaluates quality of life in five fields including exercise ability, self-management, daily activities, pain/discomfort, and anxiety/depression. Each field is divided into three levels with 1 indicating a good state of health. The EQ-5D also includes a visual analog scale in which a subject indicates subjective and overall individual health status on a vertical scale where the best health state is 100 points and the poorest health state is 0 points [27].

The MAS assesses muscles by measuring spasticity in the wrist and elbow joints while the joints are maximally bent. Scores range from 0, where there is no increase in muscle tone, to 4, where there is contracture in bent and straight muscles and/or joints [15].

The Hand Grip Strength Test evaluates muscle strength in the hands and is conducted twice in six stages; the higher value (kg) of the two tests is recorded [9].

In the current study, MEPs are evoked by stimulating the primary motor cortex on representation of hand grip muscles without pain, and responses of the FDI muscle are then observed. MEPs are useful to predict functional recovery in CI. Here, the cerebral hemisphere is magnetically stimulated in a figure 8 shape while the patient is lying down. Stimulations increase in intensity from 0 to 100 % until the MEP with the shortest latency occurs. The latency and the amplitude of the MEP responses are recorded [28].

The mRS is a six-point, ordinal hierarchical scale that describes “global disability” with a focus on mobility. The mRS is often used in contemporary stroke studies as a measure of premorbid ability and is used to assist in the selection of patients and as a final outcome measure. The mRS uses a 5-min non-standardized interview, and has six potential scores (0–5), which describe a full range of stroke outcomes; a score of 6 denotes death [29, 30].

The 9HPT is useful in measuring dexterity in relatively well-recovered patients. In the current study, participants are instructed to place nine pegs into nine holes on a board as fast as possible. Scores are computed as pegs/s, averaged over three trials, and normalized to the average score of the unaffected hand (range 0–1; 0 = cannot do) [31].

### Incidence of adverse events

Adverse events refer to undesirable and unintentional signs, symptoms, or diseases that appear after treatment in a clinical trial. Importantly, these events may not have a causal relationship with the therapy employed; nevertheless, they should be documented. Adverse events expected from this study include nausea, vomiting, headaches, dizziness, skin irritation, convulsions, and objective worsening of existing symptoms. The CRC will record adverse events, their progress, and their causal relationship with treatments in detail and will report them to the principal investigator (PI) and Institutional Review Board (IRB). If serious adverse events occur, defined as those causing severe disability or malfunction, then appropriate measures will be taken and incidents will be immediately reported to the PI and IRB.

### Sample size

The sample size was calculated based on Cohen’s formula. As there are very few precedent studies that have conducted SAEM-CS similar to the present study, and because characteristics of the variables being tested are diverse, it was difficult to select and apply a single effect size. Thus, we set the effect size as 0.25 based on a medium *f*, which is one of Cohen’s criteria [32]. Accordingly, we have established the number of groups as 4, number of repetitions as 3, effect size as 0.25, significance level as 0.05, and statistical power as 0.8. Therefore, the total sample size required for a repeated measures ANOVA (taking into account interaction time with the treatment method), calculated using G*power, is 40 (10/group) [33].

Also, because the present study is an exploratory clinical trial, we determined that a total of 60 participants, 15 in each group, would be more appropriate. We based this on a previous study that recommended 12 participants per group for pilot studies that lack prior information [34] and other conditions used to determine the sample size for a pilot study [35] that gave a sample size of 12 per group, to which a drop-out rate of 25 % was applied. In order to minimize the dropout, the CRC will manage the treatment schedule of subjects.

Moreover, we considered the feasibility of the study, since participants will be patients with CI (which, as of 2014 average 46 per month) being treated at the Department of Neurology at the Chonnam National University Hospital. Considering the fact that among them, only 10–20 % will receive inpatient treatment at the Department of Rehabilitative Medicine and meet the selection criteria, it reaffirmed our estimation that 60 participants would be appropriate and feasible to achieve the study objectives. This also takes into account recruitment, study period, and drop-out possibilities.

### Data monitoring

The Data Monitoring Committee (DMC) is composed of the PI and coordinators who are in charge of scalp acupuncture needles (Min-yeong Song, resident of the Department of Korean Medicine Rehabilitation, College of Traditional Korean Medicine, Dong-Shin University) and rTMS (Eom-ji Kim, an occupational therapist in the Department of Physical and Rehabilitation Medicine at Chonnam National University Hospital). If needed, they will report the monitoring results to the PI. Finally, this study is independent from any sponsors.

### Data analysis

Continuous data will be presented as means and standard deviations, while categorical data will be presented as frequencies and percentages. A repeated measures ANOVA will be conducted for the FMA and all secondary outcomes (NIHSS, MBI, FIM, K-MMSE, 9HPT, ASHA-NOMS, FAC, EQ-5D, MAS, Hand Grip Strength, and MEPs). Dependent variables include measured values before and after intervention and the measured values 4 weeks after completion of intervention. An *F* test will be conducted to detect differences between therapies, and Tukey’s post hoc test will be conducted to identify the groups. Repeated contrast tests will be conducted to account for time differences in each group, and the interaction group and time test will be conducted. A *p* value of <0.05 will be considered significant, and participants who drop out of the study will be excluded from the analysis. In brief, only complete case analyses will be used; thus, only subjects who complete the three evaluations will be analyzed. All statistical analyses will be performed using SPSS version 22.0 software (SPSS Inc., Chicago, IL, USA).

Data from participants who engage in less than 70 % of protocol adherence (i.e., receive <10 treatments from 15 trials) will be eliminated. Missing values will be implemented by multiple imputations. In addition, statistical differences will be verified by comparing attribution between eliminated and completed participants to check particular factors causing drop-out.

### Confidentiality and data management

Participant identification records will be kept confidential until the results of this study are published. All documents related to a clinical trial, such as case report forms (CRFs), will be recorded and labeled with participant identification codes and will not show the name of the participant. The serial number codes will be inserted in sealed, opaque envelopes, kept in a double-locked cabinet, and opened in the presence of the patient and a guardian.

All data of the participants will be recorded in Excel files by the CRC. Additionally, raw data (CRFs) will be kept in a cabinet until the end of this study.

Written informed consent will be obtained from the participants for publication of their individual details and accompanying images in this manuscript. The consent forms will be available for review by the Editor-in-Chief.

## Discussion

This protocol, following the Standard Protocol Items: Recommendations for Interventional Trials (SPIRIT) advice and Consolidated Standards Of Reporting Trials (CONSORT) 2010 guidelines, is designed to explore the synergistic effect of SAEM-CS on the recovery of motor function in patients in the acute stage of CI [36] (Additional file 4). We expect that rTMS will enhance the effect of SA and compensate for a commonly encountered problem in SA treatment: specifically, difficulty in twirling the acupuncture needle (as these needles should be twirled more than 200 times/min).

SAEM-CS is a treatment technique that combines LF-rTMS over the M1 region hot spot (motor cortex at the contralesional hemisphere) and SA stimulation of MS6 and MS7 at the upper limb regions of the lesional hemisphere to promote optimal functioning in patients following stoke. Despite other research showing that HF-rTMS applied to the lesional hemisphere more effectively improves motor function than LF-rTMS applied to the contralesional hemisphere in the early phases of stroke [9], LF-rTMS is selected for the combined SAEM-CS approach because there is some concern about complications that can arise due to the simultaneous use of SA and rTMS in the same hemisphere, and since we cannot completely exclude the interfering effects of HF-rTMS, even with non-magnetic needles. rTMS creates a powerful magnetic field near the stimulated area; therefore, all metal materials must be removed [37]. For rTMS and SA therapies that are conducted simultaneously (during SAEM-CS), we use a KOS-92 non-magnetic steel acupuncture needle that contains a much higher percentage of nitrogen (N) than STS304N1; thus, even in high processing volumes, the needle has no magnetic properties. The same method used in the SAEM-CS approach is employed for the SA and rTMS therapies. Thus, manual stimulation and electroacupuncture are not conducted in the SA group, and LF-rTMS is applied to the contralesional hemisphere in the rTMS group.

In the current study, we select patients hospitalized within 1 month after acute stroke in order to increase the homogeneity of the experimental population. This period was also chosen to increase reliability, because the most important period of recovery falls within the acute and subacute stages of ischemic stroke [38].

We expect that SAEM-CS will improve motor function; therefore, the FMA was selected as the primary outcome measure. In order to investigate unexpected effects of SAEM-CS, other scales assessing cognitive function, activities of daily living, walking, quality of life, and stroke severity are employed as secondary outcome measures.

Among TKM and CM professionals, there is a general lack of cooperation for developing techniques aimed to treat obstinate diseases. If the efficacy and safety of the above-described SAEM-CS approach is proven, it will serve as a model for collaborative research and will promote CM and TKM professionals to fuse therapeutic techniques. One hopes that such approaches can improve the treatment rates of incurable diseases.

### Dissemination policy

We will report the final data to the Ministry of Health and Welfare through the Korea Health Industry Development Institute, and we will publish the results at the end of this study.

### Trial status

This trial is ongoing. Enrollment and trial completion are expected to be finished by the end of May 2017.

## Additional files

Additional file 1: Medical Device Clinical Trial Plan Approval number 516. (DOCX 527 kb)
Additional file 2: Informed consent materials given to participants and authorized surrogates. (DOCX 370 kb)
Additional file 3: The Institutional Review Board of Chonnam National University Hospital approval of this study (CNUH-2015-114). (DOCX 1609 kb)
Additional file 4: SPIRIT checklist. (DOCX 363 kb)

## Abbreviations

CI: Cerebral infarction
SA: Scalp acupuncture
rTMS: Repetitive transcranial magnetic stimulation
SAEM-CS: Scalp acupuncture and electromagnetic convergence stimulation
CM: Conventional medicine
TKM: Traditional Korean medicine
FMA: Fugl-Meyer Assessment
NIHSS: National Institutes of Health Stroke Scale
MBI: Modified Barthel Index
FIM: Functional Independence Measurement
MMSE: Mini-Mental State Examination
ASHA-NOMS: American Speech-Language-Hearing Association National Outcomes Measurement System
FAC: Functional Ambulation Categories
EQ-5D: European Quality of Life-5 Dimensions
MAS: Modified Ashworth Scale
MEP: Motor evoked potential
mRS: Modified Rankin Scale
9HPT: 9-Hole Peg Test
