# Supplementary material for: Scalp acupuncture and electromagnetic convergence stimulation for patients with cerebral infarction: study protocol for a randomized controlled trial
Source: Trials. 2016 Oct 11;17:490. doi: 10.1186/s13063-016-1611-y (PMC5057263; doi:10.1186/s13063-016-1611-y)
Supplement: Additional file 1: — Medical Device Clinical Trial Plan Approval number 516. (DOCX 527 kb) [file 13063_2016_1611_MOESM1_ESM.docx]

**Additional file 1**

This study was approved by the Ministry of Food and Drug Safety (MFDS) (Medical Device Clinical Trial Plan Approval #516).


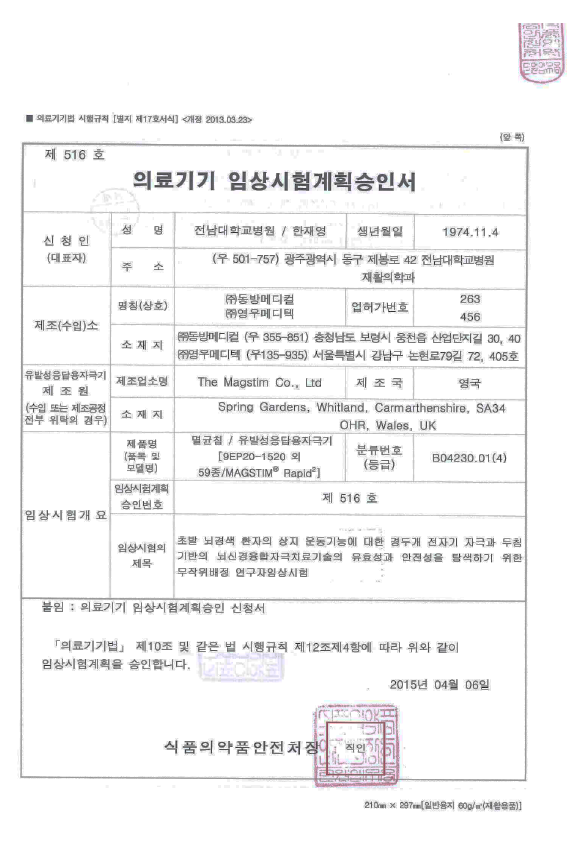


**English translations of Additional file 1**

| Article No. 516  Medical device clinical trial planning approval | | | | |
| --- | --- | --- | --- | --- |
| Applicant  (Representative) | Name | Jae-Young Han  Chonnam National University Hospital | Date of birth | 1974.11.4 |
|  | Address | (501-757) Department of Physical and rehabilitation medicine, Chonnam National University Hospital. 42, Jebong-ro, Dong-gu, Gwangju-city, Republic of Korea | | |
| Manufactorer  (Importer) | Company name | Dongbang medical Co., Ltd  Youngwoo meditech Co., Ltd | Authorization number | 263  456 |
|  | Location | Dongbang medical Co., Ltd (355-851) 30, 40, Saneopdanji-gil, Ungcheon-eup, Boryeong-si, Chungcheongnam-do, Republic of Korea  Youngwoo meditech Co., Ltd (135-935) Room 405, 72, 79-gil, Nonhyeon-ro, Gangnam-gu, Seoul, Republic of Korea | | |
| Manufacturer of  Repetitive transcranial magnetic stimulator | Company name | The Magstim Co., LTd | Manufacturing contury | United Kingdom |
|  | Location | Spring Gardens, Whitland, Carmarthenshire, SA34, OHR, Wales, UK | | |
| Summary of Clinical trial | Product name  (Model name) | Sterile acupuncture needle / Repetitive transcranial magnetic stimulator [9EP20-1520 and other 59kinds/MAGSTIM^®^ Rapid^®^] | Classification number  (grade) | B04230.01(4) |
|  | Clinical trial planning approval number | No. 516 | | |
|  | Title of clinical trial | A randomized controlled pilot trial to evaluate the effectiveness  and safety of Scalp Acupuncture and Electro-Magnetic(SAEM) Convergence Stimulation in patient with First-ever Cerebral Infarction | | |
| Attachment: Application for medical device clinical trial planning approval  In accordance with 「Medical device act」 article 10 and its enforcement rule article 12 paragraph 4  The clinical trial planning was approved as above.  April 6, 2015  **Director of Ministry of Food and Drug Safety** | | | | |
