# Supplementary material for: Scalp acupuncture and electromagnetic convergence stimulation for patients with cerebral infarction: study protocol for a randomized controlled trial
Source: Trials. 2016 Oct 11;17:490. doi: 10.1186/s13063-016-1611-y (PMC5057263; doi:10.1186/s13063-016-1611-y)
Supplement: Additional file 2: — Informed consent materials given to participants and authorized surrogates. (DOCX 370 kb) [file 13063_2016_1611_MOESM2_ESM.docx]

**Additional file 2**

Informed consent materialsgiven to participants and authorised surrogates

**1. Clinical trial**

**Scalp acupuncture and electro-magnetic convergence stimulation for patients with cerebral infarction: A study protocol for a randomized controlled trial**

You were asked to participate in this clinical study. The content below was prepared to explain to you the purpose of this research, your rights and roles. It is important that you read and understand this agreement before you agree to participate. Please take your time to read in detail and if you desire, you can discuss with family members and other people and if you have any questions, please ask the director or other research coordinators.

**2. Trial Director**

(Name) Jae-Young Han (Department) Chonnam National University Hospital (Contact) 062-220-5198

Please contact the above researcher if you have questions about the study. Also, you can reach him when you feel uncomfortable or damage yourself.

**3. Background and purpose of the clinical study**

(1) The purpose of the clinical trial

This is a clinical trial for examining the efficacy and safety of scalp acupuncture (SA) and electro-magnetic convergence stimulation (SAEM-CS), which was developed through convergence of conventional medical physicians and doctors that practice Traditional Korean medicine.

(2) Background of clinical trials

For cerebral infarction patients, transcranial electromagnetic stimulation treatment and scalp acupuncture treatment are useful treatments for motor and sensory, cognitive, language, and perception functions.

This clinical trial aims to increase therapeutic effect on cerebral infarction patients by developing “Combination of cranial nerve stimulation therapy” by combining transcranial electromagnetic stimulation and scalp acupuncture of Traditional Korean medicine. This clinical trial has not been verified of its clinical validity on humans and there are unverified experimental aspects.

**4. Information about the medical device to be used in the clinical trial**

● Repetitive Transcranial Magnetic Stimulator, (TMS)

(1) Device manufacturer: The Magstim co., Ltd

(2) Product (model) name: Repetitive Transcranial Magnetic Stimulator (A26290, 2nd grade)

(3) Product description: Repetitive Transcranial Magnetic Stimulator can be largely composed of Repetitive Transcranial Magnetic Stimulator, display unit, power supply, 70mm double coil, power cable, connection cables, foot switch, electrode connection box and stand of coil positioning guide. This is a medical device to diagnose the abnormalities in central nervous system by producing the magnetic fields and measuring the bioelectric signals with electromyogram during stimulation. Also, this device has a display function of basic evoked potentials while giving magnetic stimulation.

● Sterile needles (Nonmagnetic needles)

(1) Product manufacturer: Dongbang Medical

(2) Product (model) name: sterile needles (A84010.02 2 grade)

(3) Product description: Sterile needles (nonmagnetic needles) are used on the scalpoflesion and it is composed of a 0.25*30mm (or 0.25*40mm) sterile needle and disposable plastic sterile needle tube.

(4) Expected adverse effects and precautions

(4.1) Expected adverse effects

It means that all harmful and unintentional responses to occur during the treatment. And those are unavoidable to refer to situations which are correlated with the treatment in the clinical study. Adverse effects vary from people to people and some or all of these adverse effects can be experienced or some do not experience any adverse effects.

- The types of adverse effects expected during treatment are as follows.

Pain, bleeding or infection (redness, swelling, local pain) on acupuncture sites, hematoma, skin hypersensitive reactions, infection, headache, dizziness, nausea, vomiting, fainting

- The types of adverse effects expected during rTMS treatment are as follows.

Mild headache, redness on metal coil attachment sites , convulsion, dizziness, seizures, facial muscle contractions

When these adverse effects are happened, the research coordinator, doctor, or trial director can provide other medication to treat the pain or relief the experienced discomfort.

(4.2) Precautions for use

When applying medical devices to correspond with this clinical trial, you must follow the **selected exclusion criteria.** You must use them following the prescribed methods and rules during the supervision of doctors. If trial participants report fatigue or decrease in health status on the day of the scheduled treatment or if the research coordinator determines that the participant is not fit for treatment through observation, you can avoid potential adverse effects related treatment by adjusting the treatment date within three days before after the date.

**● Exclusion criteria**

1) When there is history of severe psychiatric disease, loss of consciousness accompanied by head trauma, brain surgery or seizure disorders

2) When there are severe medical and surgical conditions (e.g., cancer, Alzheimer’s disease, epilepsy, head trauma, cerebral palsy)

3) I magnetic stimulation is prohibited

①If there are metal substances in the head (excluding the mouth)

- Cochlea, electrodes and stimulators, aneurysm clips or coils, stents, bullet pieces, deep brain simulator, vagus nerve stimulators or eye implants

② Patients with Artificial pacemakers, defibrillators, neural stimulators, drug pumps or with intracardiac lines

③Patients with repeated convulsion symptom

④Previous craniectomy or shunt surgery

⑤Elevated intracranial pressure symptoms such as headache, vomiting, nausea

4) If there were epileptic seizures after cerebral infection

5) If there were history of stroke accompanied by a clear clinical sign

6) When scalp acupuncture stimulation is prohibited

① Scalp scarring, inflammation from scalp injury or infection in the treatment region

②Patients have inability to stop blood flow due to clotting disturbances, such as hemophilia

③There are severe serious unusual response after acupuncture needles

7) Pregnant and breast feeding mothers

8) When the subject is accommodated in a group facility such as social welfare facilities

9) Patients who disagreed with the informed consent

10) Patients scheduled for surgery within 2 weeks

11) Transient ischemic attack (TIA)

**5. Clinical research procedures and inspection (Various interventions and procedures which the participants will experience)**

The expected participation period of this clinical trial is around two months (three weeks of treatment, four weeks of outpatient visit evaluation after treatment), and the total number of participants is 60. The time consumed in each session of treatment is around 20 minutes.

If you consent to participate in this clinical trial and sign it, the following interventions and procedures are done to confirm that you are adequate for the participation in this trial.

- Demographic and sociological information, medical history and examination, vital signs

- Simplified Korean mental status examination, overall functional evaluation

If you satisfy all the inclusion criteria of the clinical trial and determine to be adequate for this study, you will be randomized into one of four groups that differ in treatment methods. The ratio of each group is 1:1:1:1 and the placement is done through chance like flipping coins by opening the envelope with a randomized order created by a computer being read. .

▸ If you are placed in the scalp acupuncture group (15 people), you will receive 15 scalp acupuncture treatments and general rehabilitation treatments for three weeks (within +3days) on the scalp at the lesion site

▸ If you are placed in the rTMS treatment group (15 people), you will receive 15 rTMS treatments and general rehabilitation treatments for three weeks (within +3days) on the hot spot of the M1 region (motor cortex at the contralesional hemisphere).

▸ If you’re placed in the SAEM-CS group (15 people), you will receive 15 combined stimulation treatments (scalp acupuncture treatment on lesion site and rTMS on the hot spot of the M1 region (motor cortex at the contralesional hemisphere) and general rehabilitation treatments for three weeks (within +3days).

▸If you’re placed in the control group, you will receive general rehabilitation treatment for cerebral infarction.

※ Scalp acupuncture treatment is done through a visit by a doctor of Gwangju Traditional Korean Medicine Hospital of Dong-Shin University, and who had more than 2 years of clinical experience.

(Corresponds to scalp acupuncture group and SAEM-CS group)

Before and after the three weeks (15 sessions) of treatment, all groups will undergo evaluation on upper and lower body function, cerebral infarction severity examination, activities of daily living performance assessment, Simplified Korean Mental State Examination, hand function agility examination, dysphagia severity evaluation, functional gait assessment, overall function evaluation, quality-of-life evaluation, muscle stiffness scale, hand grip evaluation and measurement of motor evoked potentials. Four weeks after completion of the intervention (within ±3 days), there will be evaluations over three sessions at Chonnam National University Hospital Department of Rehabilitation for upper and lower body function, cerebral infarction severity examination, activities of daily living performance assessment, hand function agility examination, dysphagia severity evaluation, overall function evaluation, quality-of-life evaluation, muscle stiffness scale, hand grip evaluation and measurement of motor evoked potentials.

All of the content above is recorded on the case records, we will ‘always’ explain about clinical trial procedures which participants and guardians are curious about. And it is possible that the research coordinator will contact you to check the presence of adverse effects.

**6. Matters related to the unverified experimental aspects of the clinical trial**

This clinical trial trying to evaluate the efficacy and safety of scalp acupuncture and electro-magnetic convergence stimulation technique has not been verified of its clinical validity on humans and there are unverified experimental aspects.

**7. Things that the participants must comply by**

You must be aware of the clinical trial perquisites adequately and carry out the trial according to the related regulations.

①Please cooperate with treatment and evaluation schedules during the trial.

②If there are other treatments you are receiving continuously before participation of the trial, please maintain it as much as possible during the trial.

③If there are changes in amount and content of drugs administered, or additional treatments during the trial period, please report in detail to the research coordinator.

**8. Dangers or discomforts participants can experience**

① Due to transcranial magnetic stimulation treatment, during or after treatment, you can experience dizziness, light headaches, nausea, vomiting, facial muscle contraction.

② Due to scalp acupuncture treatment, during or after treatment, you can experience pain around the area, acupuncture site’s bleeding or hematoma, hypersensitive skin reaction, headaches, nausea, and vomiting.

③ If it is determined that there is in danger of safety during clinical trial, your participation in this trial can be terminated.

④If you do not wish to participate in the clinical trial, your clinical research coordinator or trial director will explain the following cerebral infarction rehabilitation methods.

*Acupuncture, Traditional Korean medicine treatment, physical therapy, occupational therapy, speech therapy, etc.

**9. Anticipated benefits**

Medical benefits are not guaranteed by participating in this clinical study. However, information obtained from this study can aid in a better treatment of patients with similar diseases.

**10. Voluntary participation**

(1) The participation in this clinical study is decided upon your voluntary participation intentions. You have the right not to participate, and you can withdraw your consent. There is no penalty even if you do not participate and your decisions do not influence your treatments, and even then you have decided to participate, you can terminate the participation of the clinical study without any prejudice, disadvantage, or loss or gain at this center.

(2) The doctor who is in charge of clinical study can eliminate you from clinical study without your consent, if it is determined that it is best for you or you have violated the conditions of this trial.

**11. Compensation and treatment methods for participants when damages related to clinical trial occur**

If there are adverse reactions of the participants because of medical equipments or clinical trial procedures during the clinical trial period, it must be reported to the clinical trial director, and you can be compensated according to the ‘Agreement for compensation for victims’.

**12. Monetary compensation the subjects will receive for participating in the clinical trial**

It is expected to improve hemiplegia associated with cerebral infarction for the participation in this study. However, it is possible that there will be no improvement because the purpose of this study is to evaluate the efficacy. Medical information obtained from you will be used as useful and precious data to prove the effects of SAEM-CS treatment for cerebral infarctions. During the clinical trial, the costs of all research related scalp acupuncture, transcranial magnetic stimulation, SAEM-CS therapy, evaluations, and examination are entirely provided by the center to the hospital and the patient can receive detailed evaluation and examination for free. However, general rehabilitation treatment costs that occur are excluded when the participants stay at Chonnam National University Hospital Department of Rehabilitation(personal expense). By participating in this clinical trial, you will be provided with a return present (gift card valued 20000 KRW)on the second visit. You will be provided with a functional assessment test related return present (gift card valued 20000 KRW)on the 16th visit and you’ll be provided with 50000 KRW of transportation compensation on the 17th visit for your personal assessment. There are no additional monetary compensations.

**13. Matters expected expenses that can occur for the participants in the clinical trial**

There are no expenses you should pay to participate in the clinical trial. If the harmful and unintentional responses by participating the clinical trial are happened during the clinical trial, there will be compensation according to the Agreement for compensation for victims.

**14. Promise of confidentiality of personal information**

(1) The confidentiality of the participants are guaranteed by strictly managing all records related to this clinical study and all records of the participants are dealt with anonymously for announcement and publish, and when photos are used, they are edited so that the participant cannot be identified. However, if Ministry of Food and Drug Safety or Clinical Trials Review Committee requires the record of the participants, it can be provided.

(2) As a part of the clinical trial, your personal records, medical records, test results, and health information are collected. This information can be used by doctor in charge of clinical trial and coordinators. And your consent represents that you allowed the use of this information for the verification of reliability of procedure by government institutions and the Institutional Review Board of Chonnam National University Hospital within the hospital which supervise this research.

**15. Reasons and situations where participation in the clinical trial will be terminated**

□ The participant or legal representative of the participant withdraws the participation consent of the trial

□The participant violates inclusion criteria or responses to exclusion criteria

□ The participant or researcher violates the experiment plans

□ Severe adverse reactions occur on the participant

□ The researcher determines to stop due to adverse reactions

□ The participant requests determination of trial due to adverse reactions

□There is no data collection due to lack of evaluations after randomization

□ Treatment adherence is under 70% (If each treatment is not done within 10 times out of 15 sessions)

□ It is impossible to trail participant

□ It is determined that there are difficulties in continuing the trial

For the case above, the clinical trial director must request termination of clinical trial to the Institutional Review Board of Chonnam National University Hospital and the committee can terminate the clinical trial according to the decision.

**16. Clinical trial related inquiries contact information**

(1) You have the right to receive adequate explanation from medical staff about any questions related to clinical trial and if there are any new information that can influence your participation during the clinical trial, the medical staff will immediately inform the participant or representative of the participant about the information.

(2) If you have questions about your rights as a participant of this study, if there are problems during the trial process, or if you need additional information, please contact the clinical trial director (Chonnam National University Hospital, Jae-young Han, 062-220-5198).

(3) If you have questions about the rights of participants in this study, you can contact The Institutional Review Board of Chonnam National University Hospital (062-220-5257).

(4) You have received a copy of the study explanation and agreement.

Agreement

**Scalp acupuncture and electro-magnetic convergence stimulation for patients with cerebral infarction: A study protocol for a randomized controlled trial**

(Please check the boxes)

1. I have invested adequate time in reading the participant agreement explanation.

2. I have received adequate explanation about the purpose and detailed processes of this clinical trial from the researcher.

3. I have questioned about matters I was curious about and received adequate responses from the researcher and understood.

4. I am aware that I can refuse or terminate the participation of the clinical trial in any period during the clinical trial and that I will not receive any disadvantages regarding treatment or other matters.

5. I am aware that even if I signed this agreement the rights of the participant is not reserved or restricted.

6. I have understood that I have received copy of each the agreement explanation and agreement.

7. I was aware that I did not have to sign as long as the responses to the questions by participants are answered adequately.

8. I agreed to participate in this clinical trial under my own intentions.

9. I have adequately been informed about the necessity of personal information collection for clinical trial and have understood it, and agree provision and utilization of my various information data protected by the personal information protection law to the research institution for this clinical trial.

<Applicant>

Name: (Signature) (Date) / /

<Representative: if required>

Name: (Signature) (Date) //

Relation to applicant:

<Trial director or Delegate>

Name: (Signature) (Date) / /

**Agreement for compensation for victims**

**1. Principle**

(1) The Task manager(Gwangju Traditional Korean Medicine Hospital of Dong-Shin University, Department of Acupuncture Medicine Jae-Hong Kim, hereinafter referred to as task manager) and Clinical trial manager (Chonnam National University Hospital Department of Rehabilitation Medicine, Jae-young Han, hereinafter referred to as Clinical trial manager) compensates for physical damage (including death) of the participants

(2) If the cause of the damage is caused by clinical trial treatment, task manager and clinical trial manager compensates the clinical trial participant.

(3) Compensation is given for continuous and severe damage rather than easily treatable damages or temporary pains.

(4) If there are damages due to adverse responses due to clinical trial treatment or damages due to adverse response treatment process, compensation is given for direct damages at fault by the clinical trial treatment.

(5) If there are damages due to the clinical trial, it is compensated generally through the insurance of the test manager and clinical trial manager.

**2. Compensation is withheld for the following cases**

(1) Damages due to adverse responses caused by treatment or drugs not provided by or processed by, or supported by Task manager Gwangju Traditional Korean Medicine Hospital of Dong-Shin University, Department of Acupuncture Medicine, Jae-Hong Kim or Clinical trial manager Chonnam National University Hospital Department of Rehabilitation Medicine, Jae-young Han.

(2) Compensations for not being able to provide effects or benefits through clinical trial treatment

(3) Damages caused from violating the agreed protocol

(4) Damages caused by inattention by the subject or the guardian of the participant

**3. Criteria of compensation**

(1) The level of compensation must be adequate according to the nature of the damage, the degree, and persistence, and it must be identical to general compensations for similar damages granted by the Korean court.

(2) If there are disagreements between the guardian and institution for the level of conversation, there needs to be consultation from an expert in both parties can accept.

Task manager, Jae-Hong Kim of Gwangju Traditional Korean Medicine Hospital of Dong-Shin University, Department of Acupuncture Medicine and Clinical trial manager, Jae-young Han, of Chonnam National University Hospital Department of Rehabilitation Medicine, referring to various contents above, will take caution so that the participants do not receive any disadvantages due to this research and pledges to be responsible according to the compensation agreement if there are problems that occur due to this research.

2015 May 14

Task manager, Jae-Hong Kim, Gwangju Traditional Korean Medicine Hospital of Dong-Shin University, Department of Acupuncture Medicine

Clinical trial manager, Jae-young Han, Chonnam National University Hospital Department of Rehabilitation Medicine
