# Supplementary material for: Scalp acupuncture and electromagnetic convergence stimulation for patients with cerebral infarction: study protocol for a randomized controlled trial
Source: Trials. 2016 Oct 11;17:490. doi: 10.1186/s13063-016-1611-y (PMC5057263; doi:10.1186/s13063-016-1611-y)

**Additional file 3**

The Institutional Review Board of Chonnam National University Hospital approved this study (CNUH-2015-114)


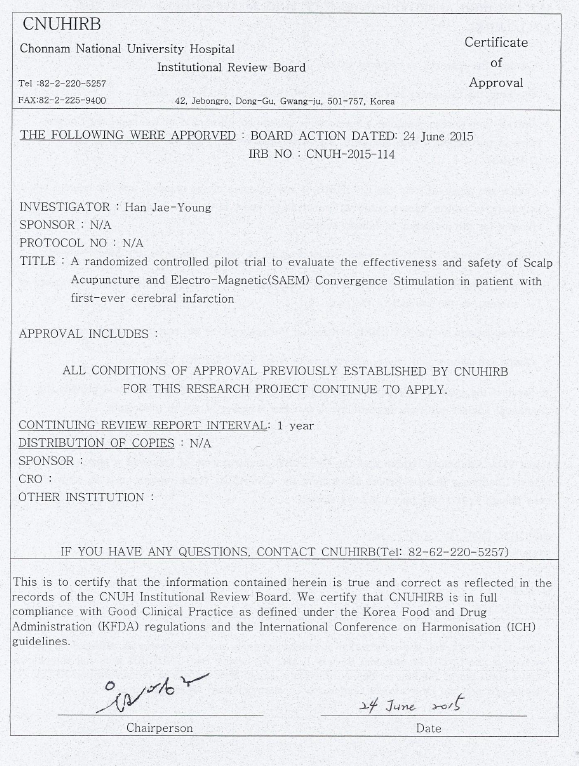


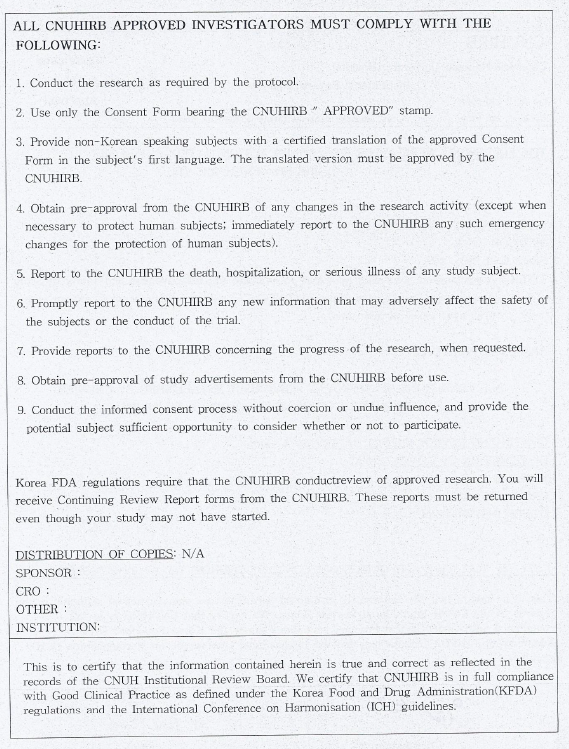

Supplement: Additional file 3: — The Institutional Review Board of Chonnam National University Hospital approval of this study (CNUH-2015-114). (DOCX 1609 kb) [file 13063_2016_1611_MOESM3_ESM.docx]
